# Ultrasensitive Quantitation of Anti-Phospholipase A2 Receptor Antibody as A Diagnostic and Prognostic Indicator of Idiopathic Membranous Nephropathy

**DOI:** 10.1038/s41598-017-12014-1

**Published:** 2017-09-21

**Authors:** Qiuhua Zhang, Biao Huang, Xiaobin Liu, Bin Liu, Yi Zhang, Zhijian Zhang, Jia Hua, Yun Fan, Ling Hu, Meijuan Meng, Mian Wu, Liang Wang, Zhigang Hu, Zhuxing Sun

**Affiliations:** 1 Wuxi People’s Hospital affiliated to Nanjing Medical University, Wuxi, 214000 China; 2 Jiangsu Institute of Nuclear Medicine, Wuxi, 214063 China

## Abstract

Anti-phospholipase A2 receptor antibody (PLA2R-Ab) is useful for affirming the diagnosis of idiopathic membranous nephropathy (IMN). Time-resolved fluoroimmunoassay (TRFIA) is highly sensitive and quantitative for measuring serum PLA2R-Ab immunoglobulin (IgG). We measured PLA2R-Ab levels with TRFIA in sera from 172 patients with IMN (n = 69), secondary MN (n = 9), and those with other glomerulonephritis (n = 94) at the time of renal biopsy compared to healthy controls (n = 286). Serum anti-PLA2R-IgG levels in healthy volunteers ranged from 0.09–0.91 mg/L. We set the cutoff value of the anti-PLA2R-IgG titer at 0.91 mg/L, with a sensitivity of 84.06% for diagnosing IMN. Increasing the cut-off value to 2.025 mg/L altered the sensitivity for diagnosing IMN to 71.01%, but with 100% specificity. IMN patients had significantly higher serum anti-PLA2R-IgG levels compared to those with secondary MN. PLA2R-Ab titers negatively correlated with estimated glomerular filtration rate (eGFR). Patinets with high titers had significantly lower serum albumin and eGFR, higher proteinuria and serum creatinine levels, accompanied by a lower complete remission rate. High titers of PLA2R-Ab were associated with poor prognosis of patients with IMN. TRFIA-based quantification of anti-PLA2R-IgG can be a reliable approach for the diagnosis and prognostication of patients with IMN.

## Introduction

In China, the percentage of patients with membranous nephropathy (MN) is increasing rapidly among those with primary glomerulopathy, from 7.1% in 2000 to 22.7% in 2009–2011^1^. MN is twice as common in men as in women, with onset occurring in patients older than 50 years of age (39.64%), and is the most common primary glomerular disease in patients over 60 years of age.

The diagnosis of MN mostly relies on kidney biopsy. Approximately 75% of MN cases are idiopathic (IMN) in origin, while others are secondary to infection (e.g. hepatitis B), autoimmune disease (e.g. lupus), medications (e.g. non-steroidal anti-inflammatory agents [NSAIDs]), and malignancies^2–4^. The diagnosis of IMN is made after excluding other known causes based on history, physical examination, laboratory tests, and microscopic examination of kidney biopsy specimens.

Previous studies showed that circulating autoantibodies against the M-type phospholipase A2 receptor (anti-PLA2R) were detectable in 52–82% of patients with IMN, but were very uncommon or absent in patients with secondary MN (SMN)^5–8^. Accordingly, anti-PLA2R antibody (PLA2R-Ab) is now accepted as a biomarker for the diagnosis of IMN due to its high sensitivity and specificity^9^, but the association of PLA2R-Ab titers with clinical features of MN, including disease activity and rate of remission, is still controversial^10–13^. This controversy may be due, in part, to the differences in assays for measuring PLA2R-Ab and in the timing of sera collection. In clinical practice, PLA2R-Ab cann’t be reliably detected because of the limitations in the measurement techniques. We used time-resolved fluoroimmunoassay (TRFIA), a new technique to measure PLA2R-Ab titers, and analyzed the correlation between PLA2R-Ab and the clinical features of IMN among a cohort of patients with glomerulopathy.

## Results

### Threshold and Analysis of Experimental Results

Samples were divided into 6 groups, including 286 healthy volunteers, 69 IMN, 55 IgA nephropathy, 16 lupus nephritis, 12 minimal change disease, and other renal diseases (3 Henoch Schonlein purpura, 4 diabetic nephropathy, 2 hepatitis B virus-associated MN, 5 arteriolonephrosclerosis, and 6 focal segmental glomerular sclerosis). Serum anti-PLA2R-IgG concentrations are shown in Table 1.

**Table 1:** The concentration of serum anti-PLA2R-IgG levels in patients with different renal diseases.

	healthy volunteers (n = 286)	IMN (n = 69)	IgA nephropathy (n = 55)	lupus nephritis (n = 16)	Minimal change disease (n = 12)	Other nephropathy (n = 20)
Means ± SD (mg/L)	0.5 ± 0.16	8.78 ± 15.54	0.9 ± 0.47	1.19 ± 0.61	1.02 ± 0.53	0.79 ± 0.42
Positive rates if anti-PLA2R-IgG > 0.91 mg/L	0	84.06%	41.82%	50%	58.33%	40%
Positive rates if anti-PLA2R-IgG > 2.025 mg/L	0	71.01%	0	0	0	0

The mean serum anti-PLA2R-IgG levels among the 286 healthy volunteers was 0.5 mg/L, ranging between 0.09 and 0.91 mg/L (mean ± 2.58 standard deviation (SD)). We then chose the cut-off value of anti-PLA2R-IgG for diagnosing kidney disease at 0.91 mg/L. Serum anti-PLA2R-IgG levels and the positive rates in the 6 groups are shown in Table 1. When the cut-off value was set at 0.91 mg/L, the positive rate (serum anti-PLA2R-IgG > 0.91 m﻿g/L) in patients with IMN was 84.06%, followed by minimal change disease (58.33%), lupus nephritis (50%), IgA nephropathy (41.82%), and other nephropathy (40%). Serum anti-PLA2R-IgG levels in patients with IMN, IgA nephropathy, lupus nephritis, minimal change disease, and other nephropathy were all significantly higher compared to the levels in the healthy volunteers (*p* = 0.000, 0.000, 0.006, 0.000, and 0.007, respectively), while serum anti-PLA2R-IgG levels in patients with IgA nephropathy, lupus nephritis, minimal change disease, and other nephropathy were significantly lower compared to the levels in the patients with IMN (*p* = 0.000 for all comparisons).

**Figure 1:**
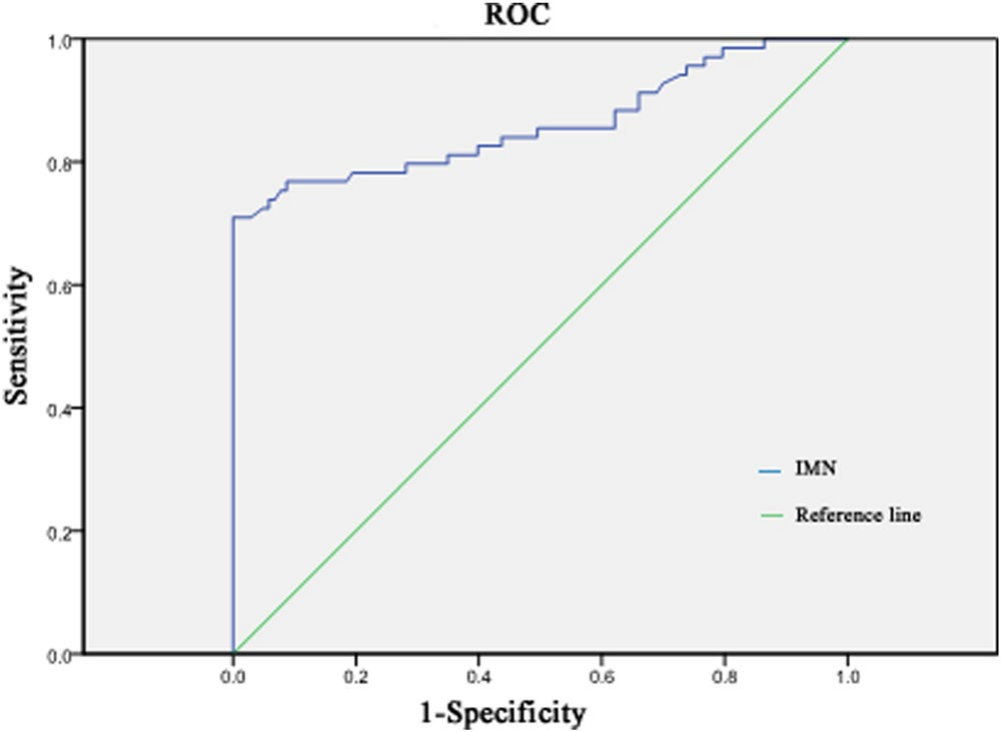
The receiver operating characteristic (ROC) curve of 2.025 mg/L as the cutoff value for distinguishing IMN from other nephropathies.

In contrast, if the cut-off value was set at 2.025 mg/L for distinguishing between IMN and other renal diseases, serum anti-PLA2R-IgG levels in patients with IgA nephropathy, lupus nephritis, minimal change disease, and other renal diseases were all lower than the threshold, while 49 of 69 patients with IMN were positive. The sensitivity of using 2.025 mg/L for diagnosis was 71.01%, with a specificity of 100% (Fig. 1).

### Receiver-operating-characteristic (ROC) Curve Analysis

**Figure 2:**
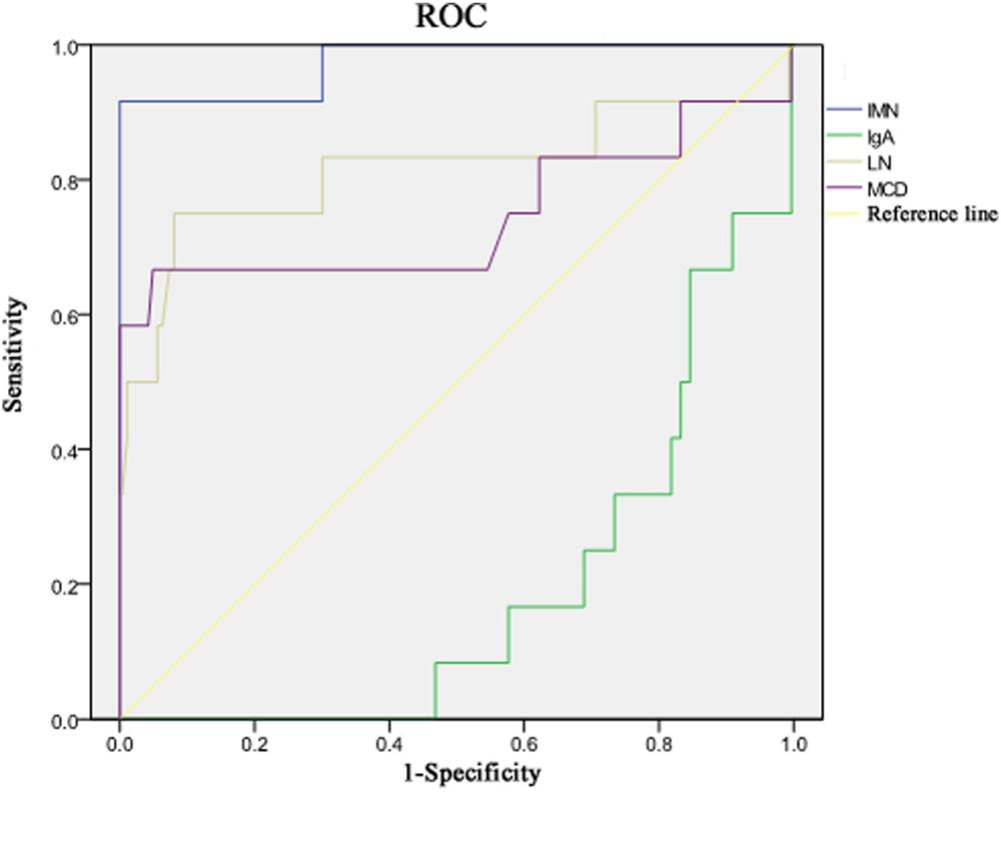
The receiver operating characteristic (ROC) curve for distinguishing different renal diseases.

We used ROC curve (AUC) analysis of serum anti-PLA2R-IgG to distinguish IMN(0.975) from IgA nephropathy (0.191), lupus nephritis (0.815), minimal change disease (0.745). The ROC curves for different renal diseases are shown in Fig. 2.

### IMN and SMN

The mean serum anti-PLA2R-IgG levels among patients with IMN were 8.78 ± 14.54 mg/L, while those among patients with SMN were 0.99 ± 0.51 mg/L. Serum anti-PLA2R-IgG levels in patients with IMN were significantly higher than those with SMN (*p* = 0.000). Among the 69 patients with IMN, there were no significant differences between those with stage I (n = 41), stage II (n = 11), stage I to II (n = 15), and stage II to III (n = 2) with regards to serum anti-PLA2R-IgG levels (*p* = 0.609).

### The Association between Serum PLA2R-Ab and Renal PLA2R Expression

Serum and renal biopsy samples were available in 63 of the 69 patients with IMN. All serum samples were collected at the same time as the renal biopsy, and neither glucocorticoids nor immunosuppressants had been used. Among the 63 patients with available samples, 47 had positive serum PLA2R-Ab levels and PLA2R immunostaining in renal biopsy specimens, 6 were serum-positive and kidney-negative, 6 were serum-negative and kidney-positive, and 4 were negative in both serum and kidney. The sensitivities of the serum PLA2R-Ab levels and renal biopsy PLA2R immunostaining were 84.13% and 80.95%, respectively. These findings are summarized in Table 2 (p < 0.05).

**Table 2:** Expression levels of serum PLA2R-Ab and Renal PLA2R staining.

Number of Patients	Serum PLA2RAb	Renal PLA2R staining
47	+	+
6	+	−
6	−	+
4	−	−
**63**	**+53**	**+51**

### Correlation with Clinical Characteristics

**Figure 3:**
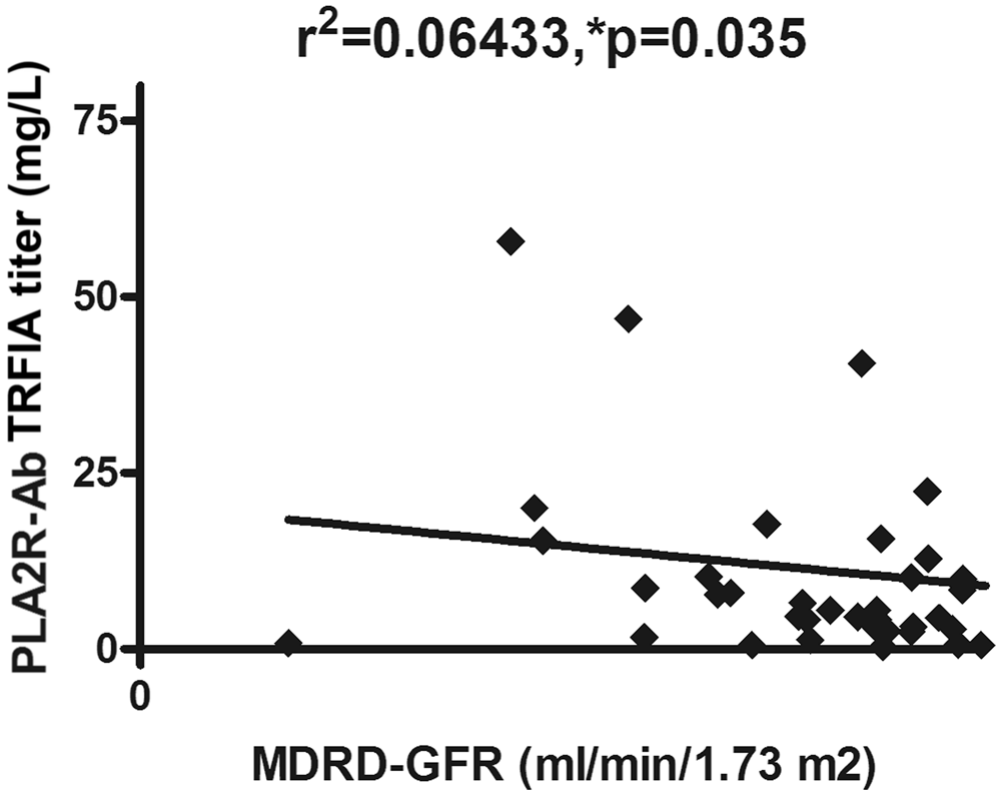
Serum PLA2R-Ab titers correlated negatively with MDRD-eGFR levels.

PLA2R-Ab titers correlated negatively with eGFR, as calculated by the Modification of Diet in Renal Disease formula (MDRD-eGFR; r = −0.254, *p* = 0.035; Fig. 3).

Patients with positive PLA2R-Ab levels (>0.91 mg/L) differed significantly from those with negative PLA2R-Ab levels (<0.91 mg/L) with regards to clinical features, including 24 hour proteinuria (4.56 *vs*. 3.03 g/day, *p* = 0.013) and serum albumin (21.45 *vs*. 26.21 g/L, *p* = 0.025) (Table 3).

**Table 3:** Clinical features of patients with positive and negative serum PLA2R-Ab levels.

Characteristics	PLA2R-Ab (+)	PLA2R-Ab (−)	p value
Number of patients	58	11	
Sex, male: female	32:26	7:4	0.61
Age	55.38 ± 12.6	43.36 ± 14.06	0.036
PLA2R-Ab titer, mg/L	10.33 ± 15.4	0.61 ± 0.14	<0.001
Serum creatinine, µmol/L	73.18 ± 20.87	96.2 ± 83.68	0.385
MDRD-eGFR (ml/min/1.73 m^2^)	86.92 ± 25.22	84.3 ± 30.6	0.695
Serum total cholesterol (mmol/L)	6.86 ± 2.41	5.99 ± 1.61	0.254
Serum triglyceride (mmol/L)	2.51 ± 1.61	2.22 ± 0.8	0.564
Serum albumin (g/L)	21.45 ± 6.3	26.21 ± 6.58	0.025
24 hours urinary protein (g/d)	4.56 ± 1.85	3.03 ± 1.59	0.013
Course of disease (months)	3.8 ± 4.3	7.72 ± 17.66	0.48
Systolic blood pressure (mmHg)	134.48 ± 17.98	130 ± 21.45	0.465
Diastolic blood pressure (mmHg)	80.16 ± 10.71	80.91 ± 9.44	0.828
Patients with nephrotic Syndrome (%)	68.97%	45.45%	0.137

### TRFIA-determined Serum PLA2R-Ab titers Associate with Poor Prognosis among Patients with IMN

To evaluate the association between serum PLA2R-Ab titers and clinical features, as well as clinical outcomes, we divided patients into high (n = 41) and low (n = 17) serum PLA2R groups, based on the 2.025 mg/L cut-off point determined by TRFIA. Patients with high serum PLA2R-Ab titers had significantly lower serum albumin (30.93 *vs*. 35.80 g/L, *p* = 0.048) and MDRD-eGFR (77.76 *vs*. 107.67 ml/min/1.73 m^2^, *p* = 0.005), and higher 24 hour proteinuria (2.34 *vs*. 1.22 g/day, *p* = 0.031) and serum creatinine (87.87 *vs*. 67.64 µmol/L, *p* = 0.049) levels, compared to those patients with low titers. Patients with high titers also had significantly lower clinical remission (51.22 *vs*. 82.35%, *p* = 0.027) and complete remission rates (47.62% *vs*. 64.29%) compared to those patients with low titers (Table 4).

**Table 4:** Clinical features of patients with high and low serum PLA2R-Ab levels.

Characteristics	PLA2R-Ab > 2.025 µg/L	PLA2R-Ab < 2.025 µg/L	p value
Number of patients	41	17	
PLA2R-Ab titer (mg/L)	10.83 ± 12.39	0.92 ± 0.47	0.000
Serum creatinine (µmol/L)	87.87 ± 36.68	67.64 ± 22.29	0.049
MDRD-GFR (ml/min/1.73 m^2^)	77.76 ± 26.80	107.67 ± 50.69	0.005
Serum albumin (g/L)	30.93 ± 8.76	35.80 ± 7.15	0.048
24 hours urinary protein (g/d)	2.34 ± 1.8	1.22 ± 1.59	0.031
Immunosuppressant use (%)	35 (85.37%)	11 (64.71%)	0.031
Remission (%)	21 (51.22%)	14 (82.35%)	0.027
Complete remission	10 (47.62%)	9 (64.29%)	
Partial remission	11 (52.38%)	5 (35.71%)	

## Discussion

The diagnosis of MN mainly relies upon clinical features and renal pathological changes. Renal biopsy is the golden criterion for diagnosing MN, but it can entail devastating sequel such as massive retroperitoneal bleeding and cannot be repeated frequently. Moreover, there are patients with contraindications to renal biopsy. Consequently, many patients with MN refuse to receive renal biopsy repetitively, and this phenomenon leads to a lack of evidence linking our therapeutic effect with patient prognosis. The anti-PLA2R-Ab can be detected in a majority of patients with IMN, and its titer is associated with disease activity and long-term outcomes^6,7,14,15^. Therefore, it is important to identify a more sensitive and accurate method for testing anti-PLA2R-Ab, and TRFIA would be a major breakthrough for patients with IMN^16^. With regard to the contemporary assays, western blot is technically complicated and not suitable for large-scale application, while the immunofluorescence test (IFT) is observer-dependent and only semi-quantitative. The detection range of enzyme-linked immunosorbent assay (ELISA) is comparatively narrow, with a detection limit of 10^−10^ mol/L, but the detection limit of TRFIA can be as low as 10^−18^ mol/L. Consequently, the sensitivity and the range of detection using TRFIA for testing anti-PLA2R-Ab can be better than those using ELISA^17^.

Serum titers of anti-PLA2R-IgG-TRFIA of IgA nephropathy, lupus nephropathy patients, minimal change nephropathy, and other nephropathy were all significantly decreased compared to IMN. The AUC of serum Anti-PLA2R-IgG used for the diagnosis of IMN was 0.975, which was significantly higher than 0.5. This indicates that serum anti-PLA2R-IgG is an important auxiliary diagnostic biomarker for distinguishing patients with IMN from those patients with other renal diseases. The AUC of sensitivity of anti-PLA2R-IgG in IMN, IgA nephropathy, lupus kidney disease, and minimal change nephropathy were 0.975, 0.191, 0.815 and 0.745, respectively. The accuracy of serum PLA2R-IgG for diagnosing different renal diseases was higher for IMN, followed by lupus nephritis, minimal change disease, and IgA nephropathy, based on their AUC values. The mean serum anti-PLA2R-IgG levels in patients with IMN was significantly higher compared to the levels in those patients with SMN, further suggesting that serum anti-PLA2R-IgG is an important auxiliary biomarker for distinguishing between IMN and SMN.

Using electron microscopy, Churg and Ehrenreich described four ultrastructural stages of MN according to the presence of subepithelial immune-complex deposit. We found that serum anti-PLA2R-IgG levels did not differ between patients with MN of different stages; thus, it is likely that no association exists between serum anti-PLA2R-IgG levels and the presence of subepithelial immune-complex deposit.

In 2009, serum PLA2R autoantibody was found only in IMN but not in other renal diseases^5^; however, more recently, PLA2R has been found in patients with SMN, such as type V lupus nephritis, hepatitis B virus-related MN, and tumor related MN^18^. When the cut-off value of anti-PLA2R-IgG concentration was set at 0.91 mg/L, patients had a 84.06% positive rate of IMN, 41.82% IgA nephropathy, 50% lupus nephritis, 58.33% minimal change disease, and 40% other renal diseases. If the cut-off value was set at 2.025 mg/L, the positive rate of patients with IMN was 71.01%, with a specificity of 100%. The threshold value for diagnosing renal diseases was 0.91 mg/L; those with serum anti-PLA2R-IgG higher than 0.91 were more likely to have renal diseases. However, those with serum anti-PLA2R-IgG levels between 0.91 to 2.025 mg/L can have a variety of renal diseases, since positive results have been observed in patients with IgA nephropathy, lupus nephritis, minimal change disease, and IMN. The positive rates of serum anti-PLA2R-IgG in patients with IMN reached 100% when the cutoff value was set at 2.025 mg/L or higher.

Of the 63 patients with detectable serum PLA2R-Ab, 47 had positive PLA2R immunostaining in glomerular deposits, while 6 patients who had high circulating PLA2R-Ab levels did not have any detectable glomerular PLA2R staining. These findings suggest that this antibody might not be nephritogenic, or that epitopes were poorly accessible at the time of renal biopsy. Six patients did not have detectable serum PLA2R-Ab, but had PLA2R glomerular deposits. This discrepancy might result from a rapid clearance of antibodies from the circulation with a rapid deposition in glomeruli, or it could be due to a late referral of patients with persistent proteinuria because of irreversible ultrastructural changes. Alternatively, assay variability may also contribute to the discrepancy in the data^19^.

We found that serum PLA2R-Ab titers were negatively correlated with MDRD-eGFR; when patients were further divided based on the cut-off value of 0.91 µg/L, as determined by TRFIA, those patients with positive findings had significantly more proteinuria and more severe hypoalbuminemia. Similarly, when we divided patients based on the cut-off value of 2.025 µg/L, those patients with higher titers had more severe disease activity and a lower rate of remission. This finding suggests that a higher titer of serum PLA2R-Ab for any given patient may signify a worse prognosis, and PLA2R-Ab can be regarded as an important prognostic factor in patients with IMN^10^.

Serum PLA2R-Ab levels measured by TRFIA can contribute to the diagnosis of IMN. The PLA2R titers can reflect disease activity and correlate closely with the rate of clinical remission. Therefore, the assessment of serum PLA2R-Ab using TRFIA may be a convenient and valuable method for predicting patient prognosis. In conclusion, TRFIA-based quantification of anti-PLA2R-IgG can be a reliable assay for the diagnosis and prognostication of patients with IMN.

## Materials and Methods

### Patient Participation

Serum samples were collected from 286 healthy volunteers without evidence of renal, gastroduodenal, or hepatic diseases at the Jiangyuan Hospital in China. Serum samples were collected from 172 patients with renal diseases at the Affiliated Wuxi People’s Hospital of Nanjing Medical University in China. Each patient underwent a renal biopsy at the time of blood sampling, and no patient received immunosuppression before being included in this study. Hypertension and proteinuria medications were continued. The renal disease patient groups presented with IMN (69), IgA nephropathy (55), lupus nephritis (16; 7 patients presented with class 5 lupus nephritis), minimal change disease (12), focal segmental glomerular sclerosis (6), arteriolonephrosclerosis (5), diabetic nephropathy (4), Henoch Schonlein purpura (3), and hepatitis B virus-associated MN (2).

All clinical investigations were conducted according to the 2008 Declaration of Helsinki and good clinical practice guidelines. Written informed consent was obtained from all subjects. The study protocols were approved by the Research Ethics Committee of the First Affiliated Wuxi People’s Hospital of Nanjing Medical University and Jiangsu Institute of Nuclear Medicine, People’s Republic of China.

### Clinical Data

We measured serum creatinine, albumin, total cholesterol, and calculated estimated glomerular filtration rate (eGFR) from the collected sera. Urine was collected at the time of renal biopsy to assess proteinuria levels using the average of three 24 hour urinary protein measurements. Patients were followed up for at least one year. Clinical data were recorded upon patient enrollment and after immunosuppressive treatment one year later. Patients were classified as having complete remission if they had proteinuria <0.3 g/24 h, normal serum albumin, and creatinine levels; patients were classified as having partial remission if they had proteinuria <3.5 g/24 h, a 50% reduction from their baseline levels, and an improvement of serum albumin and creatinine, based on the Kidney Disease Improving Global Outcomes (KDIGO) 2012 guideline.

### Renal Pathological Examination and PLA2R Immunostaining

We used light microscopy, immunofluorescence (IF), and electron microscopy to evaluate renal biopsy specimens. Direct IF for IgG, IgA, IgM, C3, C4, and C1q were done on frozen tissue sections, with results presented semi-quantitatively from 0 to 4+. Electron microscopy identification of dense deposits was semi-quantitatively defined as 0 to 3+.

Renal PLA2R detection by immunostaining of the glomeruli was performed on paraffin-embedded biopsy samples. Citrate buffer of pH 6.0 was microwaved at 100% power for 8 minutes, followed by antigen retrieval. Bovine serum albumin (3%) was used for blocking. Anti-PLA2R-Ab (Sigma, HPA012657) was diluted at 1:500 and incubated overnight at 4 °C. The secondary fluorescein Cy3-conjugated donkey anti-rabbit IgG antibody (Jackson, 711-165-152) was diluted at 1:200. Positive PLA2R staining was characterized by granular staining along the capillary loops, ranging from 0 to 3+. Negative control (secondary antibody only) was used in all staining to exclude the cross-reactions between the secondary antibody and human IgG.

### Time-Resolved Fluoroimmunoassay for PLA2R-Ab

Recombinant PLA2R-Ab was generated by cloning in 293 T cells. Goat anti-human IgG antibodies (Jackson Immuno Research, USA) were labeled with Eu3+. The optimal coating concentration of PLA2R antigen was approximately 5 μg/mL. The addition of a fluorescence intensifier enhanced the original fluorescence by 1 million times and improved the sensitivity and the range of detection during TRFIA for measuring anti-PLA2R-IgG. AutoDELFIA1235 (Perkin Elmer, USA) was used to read Eu3+ fluorescence in microtiter wells.

The measurement range of anti-PLA2R-IgG by TRFIA was 0.02–340 mg/L. The intra-assay and inter-assay coefficients of variation (CV) of anti-PLA2R-IgG by TRFIA were 3.2% and 5.6%, respectively.

### Statistical Analysis

Statistical analysis was done using the SPSS software version 18 (SPSS, Inc., Chicago, Ill., USA). Data are presented as mean ± standard error (SE). Correlation analysis was done using the Pearson’s correlation. Statistical significance was set at *p* < 0.05.

## References

[1] Pan X (2013). Changing Spectrum of Biopsy-Proven Primary Glomerular Diseases Over the Past 15 Years: A Single-Center Study in China. CONTRIB NEPHROL..

[2] Feng Z (2016). A Follow-Up Analysis of Positron Emission Tomography/Computed Tomography in Detecting Hidden Malignancies at the Time of Diagnosis of Membranous Nephropathy. ONCOTARGET..

[3] Zeng CH (2008). Etiology and Clinical Characteristics of Membranous Nephropathy in Chinese Patients. AM J KIDNEY DIS..

[4] Lefaucheur C (2006). Membranous Nephropathy and Cancer: Epidemiologic Evidence and Determinants of High-Risk Cancer Association. KIDNEY INT..

[5] Beck LJ (2009). M-Type Phospholipase A2 Receptor as Target Antigen in Idiopathic Membranous Nephropathy. N Engl J Med..

[6] Hofstra JM, Beck LJ, Beck DM, Wetzels JF, Salant DJ (2011). Anti-Phospholipase A(2) Receptor Antibodies Correlate with Clinical Status in Idiopathic Membranous Nephropathy. Clin J Am Soc Nephrol..

[7] Hoxha E (2011). An Immunofluorescence Test for phospholipase-A(2)-receptor Antibodies and its Clinical Usefulness in Patients with Membranous Glomerulonephritis. Nephrol Dial Transplant..

[8] Segarra-Medrano A (2014). Prevalence, Diagnostic Value and Clinical Characteristics Associated with the Presence of Circulating Levels and Renal Deposits of Antibodies Against the M-type Phospholipase A2 Receptor in Idiopathic Membranous Nephropathy. NEFROLOGIA..

[9] Dou Y (2016). The Accuracy of the Anti-Phospholipase A2 Receptor Antibody in the Diagnosis of Idiopathic Membranous Nephropathy: A Comparison of Different Cutoff Values as Measured by the ELISA Method. INT UROL NEPHROL..

[10] Kim YG (2015). Anti-Phospholipase A2 Receptor Antibody as Prognostic Indicator in Idiopathic Membranous Nephropathy. AM J NEPHROL..

[11] Wei SY (2016). Serum Anti-PLA2R Antibody Predicts Treatment Outcome in Idiopathic Membranous Nephropathy. AM J NEPHROL..

[12] Lin W (2015). the Relationship Between Anti-Phospholipase A2 Receptor Antibody and Idiopathic Membranous Nephropathy. Zhonghua Nei Ke Za Zhi..

[13] Ruggenenti P (2015). Anti-Phospholipase A2 Receptor Antibody Titer Predicts Post-Rituximab Outcome of Membranous Nephropathy. J AM SOC NEPHROL..

[14] Kanigicherla D (2013). Anti-PLA2R Antibodies Measured by ELISA Predict Long-Term Outcome in a Prevalent Population of Patients with Idiopathic Membranous Nephropathy. KIDNEY INT..

[15] Akiyama S (2015). Prevalence of Anti-Phospholipase A2 Receptor Antibodies in Japanese Patients with Membranous Nephropathy. CLIN EXP NEPHROL..

[16] Huang, B. *et al*. Improvement of Idiopathic Membranous Nephropathy Diagnosis with Ultrasensitive Quantitative Detection of Anti-Phospholipase A2 Receptor. J Allergy Clin Immunol. (2016). 10.1016/j.jaci.2016.10.020 27876629

[17] Huang B (2017). A Novel Time-resolved Fluoroimmunoassay for the Quantitative Detection of Antibodies Against the Phospholipase A2 Receptor. Sci Rep..

[18] Qin W (2011). Anti-Phospholipase A2 Receptor Antibody in Membranous Nephropathy. J AM SOC NEPHROL..

[19] Debiec H, Ronco P (2011). PLA2R Autoantibodies and PLA2R Glomerular Deposits in Membranous Nephropathy. N Engl J Med..

